# Evaluation and management of symptomatic duodenal diverticula: a single-center retrospective analysis of 647 patients

**DOI:** 10.3389/fsurg.2023.1267436

**Published:** 2023-08-30

**Authors:** Jiaqiang Ren, Jiachun Ding, Tong Su, Shuai Wu, Fan Chen, Jie Li, Zheng Wang, Liang Han, Zheng Wu

**Affiliations:** ^1^ Department of Hepatobiliary Surgery, The First Affiliated Hospital of Xi'an Jiaotong University, Xi'an, China; ^2^ School of Public Health, Xi'an Jiaotong University Health Science Center, Xi'an, China

**Keywords:** duodenal diverticula, symptomatic patients, diverticular size, biliary comorbidities, surgical treatment

## Abstract

**Aims:**

To explore the clinical characteristics of patients with symptomatic duodenal diverticula and to generalize how to make appropriate treatment choices for this group of patients.

**Materials and methods:**

From January 2010 to September 2020, a total of 647 patients with duodenal diverticula (DD) were included in this study. 345 of them with relevant symptoms were divided into the symptomatic group and the other 302 patients were in the asymptomatic group.

**Results:**

Among all patients, most DD were located in the periampullary area, <1 cm in size, and single in number. The distribution of DD localized in the 2nd portion/periampullary (*P* = 0.002/*P* < 0.001) and with a 1 cm size cut-off value (*P* = 0.003) was significantly different between the symptomatic and asymptomatic groups. Multivariate Logistics analysis further suggests that diverticular size (<1 cm, 1–3 cm) and combined biliary comorbidities (bile duct stones and gallstones, primary bile duct stones, cholangitis without bile duct stones) may be factors influencing the choice of treatment modality. Of all patients undergoing surgical treatment, a total of 7 cases developed various postoperative complications, and no one died.

**Conclusions:**

Patients with DD ≥1 cm or located in the periampullary were more likely to be symptomatic. The specific size of the DD and the combination of specific biliary comorbidities may have an impact on the choice of treatment modality.

## Introduction

Diverticulum is usually manifested as part of the intestinal wall structure herniated out due to the muscular layer defect, forming a pouch structure (1). Duodenal diverticula (DD) is one of the most common digestive tract diverticula (2), which was first discovered and reported by French pathologist Chomel. There are 70%–75% of DD that occurs in the radius of 2–3 cm around the ampullary of Vater (3), so it is also called periampullary duodenal diverticula (PAD). Detection rates for PAD under endoscopic retrograde cholangiopancreatography (ERCP) ranged from 5.1% to 32.8% (4–8), and from 23.0% to 32.0% at autopsy (9). PAD is more common among middle-aged and elderly people, and there is no obvious gender difference.

Generally, no special treatment is required for patients with asymptomatic DD. Only about 5% (10) of DD patients may show symptoms such as epigastric pain, nausea, and vomiting due to diverticulitis or diverticular perforation/hemorrhage. DD, especially PAD, may be associated with various biliopancreatic complications such as choledocholithiasis, pancreatitis, and common bile duct obstruction, and may accompany various relevant symptoms. Such as Lemmel's syndrome (11), which is defined as obstructive jaundice due to a PAD in the absence of choledocholithiasis or a neoplasm. At present, the studies on DD mostly focus on its association with biliopancreatic diseases or endoscopic therapy (12–14), while there are few reports on when and how to deal with symptomatic DD patients. Therefore, we designed this retrospective study aims to explore the clinical characteristics of symptomatic patients and to generalize how to make appropriate treatment choices for this group of patients.

## Materials and methods

This study was approved by the Ethics Committee of the First Affiliated Hospital of Xi'an Jiaotong University.

**Figure 1:**
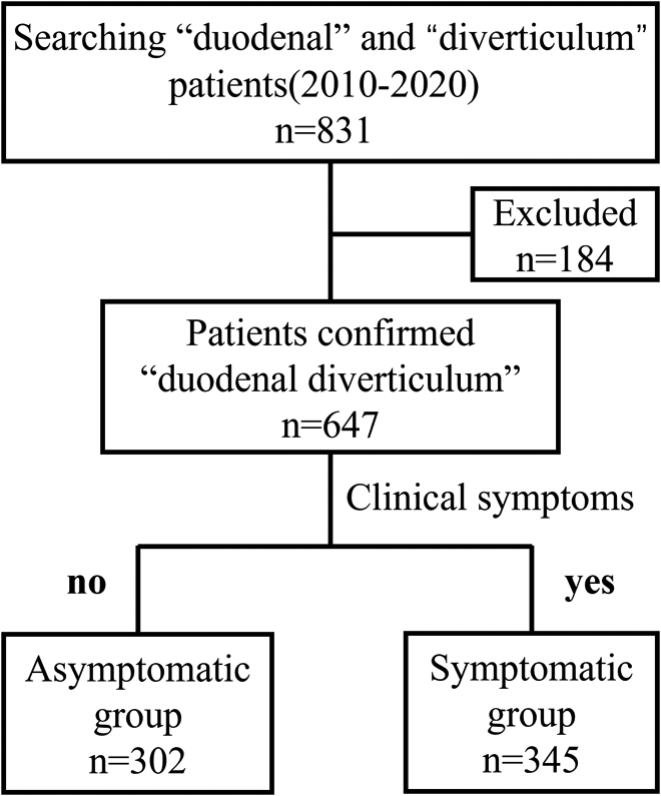
Flow diagram of inclusion in the study.

Patients with radiographic findings or clinical diagnosis of DD were searched through the PACS (picture archiving and communication system) and the medical record system for a decade (from January 2010 to September 2020), and a total of 831 patients were initially identified. Then we retrieved and recorded all patients' clinical data one by one with the hospital ID number identification, including basic characteristics like (1) name, sex, age, and other baseline characteristics; (2) the location, size, and number of DD; (3) clinical signs and symptoms, complications, and other positive imaging examinations; (4) treatment modalities and outcomes, etc. Patients who met one or more of the following exclusion criteria were excluded: (1) outpatients or those without complete data of hospitalization; (2) patients with clinically confirmed pseudo-DD (Ulcers, artifacts, etc.); (3) lack of reliable imaging data to confirm the existence of DD; (4) repeated patients. Figure 1 showed the Flow diagram of inclusion in the study.

All imaging data (Supplementary Figure S1**)** were retrieved from the picture archiving and communication system (PACS) of our hospital. The size of DD was evaluated on coronal reconstructed images at the PACS monitor using electronic calipers, and the size of the DD is based on the longest dimensions measured. To minimize the error caused by human factors as much as possible, two researchers independently conducted the data collection and collation stage, and finally summarized the data after comparison.

Statistical analysis was performed using SPSS 22.0 software (SPSS, Chicago, IL, USA). Measurement data with normal distribution were expressed as mean ± standard deviation while the non-normal distribution data are described by the median, and a *t*-test was used for comparison. The counting data were expressed as the number of cases and percentage, and the chi–square test was used for comparison. Univariate analysis and multivariate analyses (logistic regression) were used to judge the association between various parameters and specific outcome events. *P* value < 0.05 was considered statistically significant.

## Results

After excluding 184 patients based on the exclusion criteria, a total of 647 patients with DD were enrolled in this study. And 345 patients with all kinds of relevant symptoms were divided into the symptomatic group, the remaining 302 patients were in the asymptomatic group. Patients in the symptomatic group were divided into different subgroups according to the treatment modality, and those who received surgical treatment were further differentiated by different procedures.

**Figure 2:**
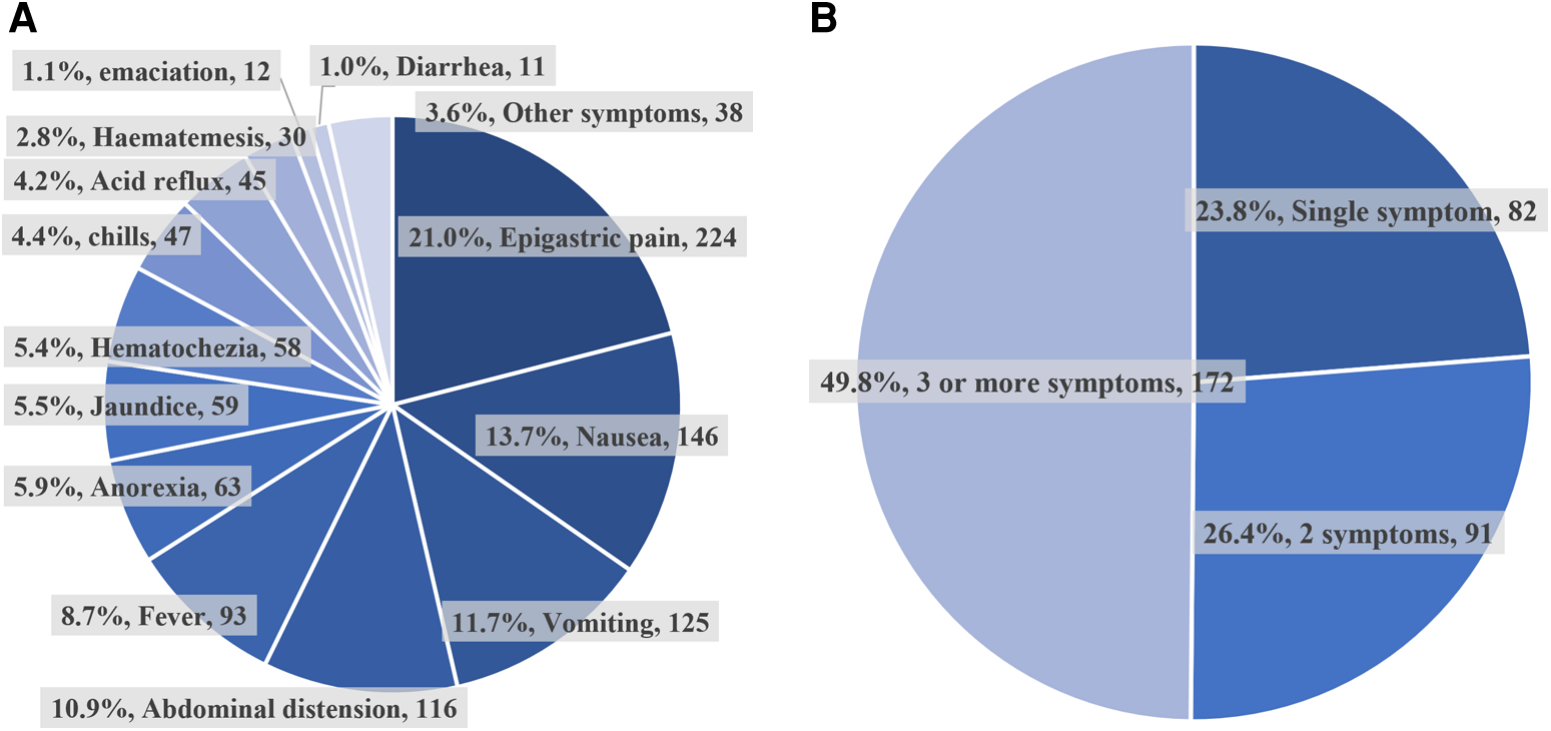
The ratio of different symptomatic types and numbers. (**A**) The percentage of different clinical symptoms in the symptomatic group. (**B**) The percentage of patients with a different number of symptoms in the symptomatic group.

Of all 345 patients in the symptomatic group, the percentage of their clinical symptoms and the percentage of patients with a different number of comorbid symptoms were shown in Figure 2. The most common symptoms of them were epigastric pain (21.2%), next by nausea (13.8%), vomiting (11.8%), abdominal distension (11.0%), etc. Nearly half of the patients (172, 49.8%) had 3 or more symptoms at the same time, and the fewest patients (82, 23.8%) had a single symptom.

### Differences in baseline characteristics between patients with DD in the symptomatic and asymptomatic groups

Among all patients 359 (55.5%) were male and 288 (44.5%) were female, with a male-to-female ratio of 1.25:1. The mean age of all symptomatic group patients was younger than that of the asymptomatic group (60.01 ± 13.79 vs. 61.12 ± 13.43 years, *P* = 0.303). And the majority of patients with DD were first diagnosed in the 40–80 age group, both in the symptomatic group and asymptomatic group (Table 1).

**Table 1:** Distribution of diverticular patient in baseline characteristics between the symptomatic and asymptomatic groups.

	Symptomatic group (*n* = 345)	Asymptomatic group (*n* = 302)	*P*-value
Basic characteristics			
Sex, *n* (%)			0.700
Male	189 (54.8)	170 (56.3)	
Female	156 (45.2)	132 (43.7)	
Age, (years, mean ± SD)	59.93 ± 13.82	61.03 ± 13.25	0.303
Age groups, *n* (%)			
0–20	4 (1.2)	4 (1.3)	0.850
21–40	26 (7.5)	17 (5.6)	0.331
41–60	126 (36.5)	107 (35.4)	0.773
61–80	179 (51.9)	165 (54.7)	0.484
>80	10 (2.9)	9 (3.0)	0.951
Diverticular characteristics			
Location, *n* (%)			
1st portion	34 (9.9)	36 (11.9)	0.399
2nd portion	221 (64.1)	158 (52.3)	0.002
Periampullary	178 (51.6)	113 (37.4)	<0.001
Non-periampullary	43 (12.5)	45 (14.9)	
3rd portion	39 (11.3)	41 (13.6)	0.381
4th portion	8 (2.3)	13 (4.3)	0.155
Multiple in single portion^a^	15 (4.3)	20 (6.6)	0.202
Multiple in multi-portions^b^	19 (5.5)	21 (7.0)	0.446
Multiple inside and outside the duodenum^c^	9 (2.6)	13 (4.3)	0.482
Number, *n* (%)			
Single	297 (86.1)	251 (83.1)	0.294
Doube	38 (11.0)	44 (14.6)	0.175
Multiple (≥3)	10 (2.9)	7 (2.3)	0.645
Size, *n* (%)^d^			
<1 cm	133 (40.8)	156 (52.5)	0.003
≥1 cm and <3 cm	117 (35.9)	87 (29.3)	0.080
≥3 cm and <5 cm	54 (16.6)	41 (13.8)	0.339
≥5 cm	22 (6.7)	13 (4.4)	0.199

^a^ The presence of 2 or more diverticula in one portion of the duodenum.

^b^ The presence of diverticula in at least 2 portions of the duodenum.

^c^ The presence of diverticula in any one or more organs other than duodenum.

^d^ The size of diverticula was measured with electronic calipers in the imaging images, and patients who did not have effective and measurable imaging data were not included in size counting.

The most common location of DD was the 2nd portion, which showed a statistically significant difference between the two groups (*P* = 0.002). Also, the distribution of patients with PAD was statistically significant between the two groups (*P* < 0.001). We have also found that the majority of patients (84.7%) present with only single DD, with three or more DD occurring very rarely (2.6%). As for the size of DD, more patients (44.7%) had diverticula <1 cm in diameter. Further, the distribution of DD <1 cm and ≥1 cm was significantly different (*P* = 0.003) in the symptomatic and asymptomatic groups. Therefore, our results suggest that the location and size of a particular DD may be critical to the presence of symptoms.

### Distribution of different biliopancreatic comorbidities and diverticular complications in symptomatic patients with 2nd portion DD

Given the significant differences in the distribution of DD located in the 2nd portion, periampullary, and with a cut-off value of 1 cm in size between the symptomatic and asymptomatic groups. To further corroborate this finding, we further explored the possible clinical sources of symptoms in patients with DD in specific sites and sizes, as the combination of various biliopancreatic comorbidities and diverticular complications.

Our results show that various comorbidities and complications seem to be more frequent in the group of DD ≥1 cm, especially when the PAD and ≥1 cm (Table 2). Some biliopancreatic comorbidities (bile duct stones and gallstones, *P* = 0.034; primary bile duct stones, *P* = 0.045; pancreatitis without bile duct stones, *P* = 0.044) and some diverticular complications (diverticulitis, *P* = 0.008; diverticular hemorrhage, *P* = 0.026) had statistically significant differences in distribution between different groups of sites and sizes, which suggested that larger DD (≥1 cm) or/ (and) specific locations (periampullary) are more likely to have clinical manifestations that require management.

**Table 2:** Distribution of different comorbidities/complications in the symptomatic group of patients with 2nd portion diverticula.

	Periampullary <1 cm (*n* = 29)	Periampullary ≥1 cm (*n* = 149)	Non-Periampullary <1 cm (*n* = 68)	Non-Periampullary ≥1 cm (*n* = 99)	*P*-value
Biliary comorbidities, *n* (%)					
Gallbladder stones	5 (17.2)	29 (19.5)	11 (16.2)	9 (9.1)	0.176
Bile duct stones and gallstones	2 (6.9)	30 (20.1)	6 (8.8)	10 (10.1)	0.034
Primary bile duct stones	6 (20.7)	32 (21.5)	7 (10.3)	10 (10.1)	0.045
Cholangitis without bile duct stones	0	10 (6.7)	2 (2.9)	5 (5.1)	0.510
Pancreatic comorbidities, *n* (%)					
Biliary pancreatitis	2 (6.9)	17 (11.4)	5 (7.4)	3 (3.0)	0.118
Pancreatitis without bile duct stones	1 (3.4)	17 (11.4)	2 (2.9)	4 (4.0)	0.044
Diverticular complications, *n* (%)					
Diverticulitis	1 (3.4)	30 (20.1)	5 (7.4)	23 (23.2)	0.008
Diverticular perforation	0	2 (1.3)	0	1 (1.0)	–
Diverticular hemorrhage	0	1 (0.7)	2 (2.9)	7 (7.1)	0.026
Duodenal obstruction	1 (3.4)	0	0	1 (1.0)	–

### Distribution of relevant clinical characteristics among different treatment modality groups of patients in the symptomatic group

After exploring the potential factors associated with symptomatic DD, we next turn to the management of this group of patients. We summarized the management of all 345 symptomatic patients, including direct surgical treatment of DD, indirect treatment of complications (endoscopic treatment or cholecystectomy), and conservative treatment. The most common treatment is conservative treatment (225, 65.2%), followed by surgical treatment (64, 18.6%) (Table 3). Univariate analysis showed a significant difference in the distribution of variables such as diverticular size (<1 cm, *P* = 0.001; ≥ 5 cm, *P* = 0.003), diverticular location (1st portion, *P* = 0.002; 2nd portion, *P* = 0.002; periampullary, *P* < 0.001; and 3rd portion, *P* = 0.009), number of diverticula (single, *P* = 0.016; multiple, *P* = 0.004), combined biliary comorbidities (bile duct stones and gallstones, *P* < 0.001; primary bile duct stones, *P* < 0.001) and pancreatic comorbidities (biliary pancreatitis, *P* = 0.016) between the different treatment groups.

**Table 3:** Different treatment options for patients in the symptomatic group.

	Surgical treatment (*n* = 64)	Endoscopic treatment (*n* = 26)	Cholecystectomy (*n* = 30)	Conservative treatment (*n* = 225)	*P*-value
Sex, *n* (%)					0.092
Male	29 (45.3)	10 (38.5)	16 (53.5)	132 (58.7)	
Female	35 (54.7)	16 (61.5)	14 (46.7)	93 (41.3)	
Age groups, *n* (%)					
0–40	4 (6.3)	1 (3.8)	1 (3.3)	19 (8.4)	0.808
40–80	59 (92.2)	23 (88.5)	27 (90.0)	199 (88.4)	0.856
>80	1 (1.6)	2 (7.7)	2 (6.7)	7 (3.1)	0.272
Size, *n* (%)					
<1 cm	11 (17.2)	5 (19.2)	17 (56.7)	77 (34.2)	0.001
≥1 cm and <3 cm	18 (28.1)	9 (34.6)	15 (50.0)	79 (35.1)	0.231
≥3 cm and <5 cm	18 (28.1)	6 (23.1)	5 (16.7)	31 (13.8)	0.051
≥5 cm	8 (12.5)	3 (11.5)	5 (16.7)	8 (3.6)	0.003
Location, *n* (%)					
1st portion	2 (3.1)	0	0	31 (13.8)	0.002
2nd portion	48 (75.0)	22 (84.6)	14 (46.7)	133 (59.1)	0.002
Periampullary	45 (70.3)	18 (69.2)	11 (36.7)	85 (37.8)	<0.001
Non-periampullary	3 (4.7)	4 (15.4)	3 (10.0)	48 (21.3)	
3rd portion	1 (1.6)	1 (3.8)	5 (16.7)	30 (13.3)	0.009
4th portion	1 (1.6)	0	2 (6.7)	6 (2.7)	0.469
Multiple in single portion	1 (1.6)	2 (7.7)	4 (13.3)	9 (4.0)	0.054
Multiple in multi-portions	7 (10.9)	1 (3.8)	3 (10.0)	9 (4.0)	0.103
Multiple inside and outside the duodenum	4 (6.3)	0	2 (6.7)	7 (3.1)	0.328
Number, *n* (%)					
Single	51 (79.7)	23 (88.5)	21 (70.0)	201 (89.3)	0.016
Doube	11 (17.2)	1 (3.8)	5 (16.7)	21 (9.3)	0.141
Multiple (≥3)	2 (3.1)	2 (7.7)	4 (13.3)	3 (1.3)	0.004
Biliary comorbidities, *n* (%)					
Gallbladder stones	9 (14.1)	1 (3.8)	8 (26.7)	32 (14.2)	0.129
Bile duct stones and gallstones	18 (28.1)	6 (23.1)	19 (63.3)	10 (4.4)	<0.001
Primary bile duct stones	22 (34.4)	15 (57.7)	3 (10.0)	14 (6.2)	<0.001
Cholangitis without bile duct stones	7 (10.9)	2 (7.7)	1 (3.3)	14 (6.2)	0.548
Pancreatic comorbidities, *n* (%)					
Biliary pancreatitis	8 (12.5)	3 (11.5)	4 (13.3)	9 (4.0)	0.016
Pancreatitis without bile duct stones	7 (10.9)	1 (3.8)	2 (6.7)	15 (6.7)	0.632
Diverticula complications, *n* (%)					
Diverticulitis	16 (25.0)	1 (3.8)	5 (16.7)	35 (15.6)	0.088
Diverticular perforation	1 (1.6)	0	0	3 (1.3)	0.848
Diverticular hemorrhage	0	0	0	10 (4.4)	0.219
Duodenal obstruction	0	0	1 (3.3)	2 (0.9)	0.479

To further confirm the findings, a multivariate analysis of variables was performed (Table 4). The results suggest that the combination of bile duct stones and gallstones, primary bile duct stones, and cholangitis without bile duct stones may be the reason for preferring surgical treatment as well as endoscopic treatment compared to conservative treatment, while DD sizes <1 cm or 1–3 cm are the possible reason for preferring conservative treatment. The above results suggest that the options of different treatments for patients with symptomatic DD may be related to the specific diverticular sizes and biliary comorbidities.

**Table 4:** Multivariate analysis of different treatment options for patients in the symptomatic group.

Treatments	Variables	OR	95% Cl		*P*-value
			Lower bound	Upper bound	
Surgical treatment*					
	Size				
	<1 cm	0.161	0.032	0.807	0.026
	≥1 cm and <3 cm	0.2	0.045	0.883	0.034
	Biliary comorbidities				
	Bile duct stones and gallstones	34.537	8.994	132.626	<0.001
	Primary bile duct stones	26.671	7.742	91.882	<0.001
	Cholangitis without bile duct stones	4.159	1.006	17.196	0.049
Endoscopic treatment*					
	Size				
	<1 cm	0.09	0.01	0.817	0.032
	Biliary comorbidities				
	Bile duct stones and gallstones	30.76	4.3	220.056	0.001
	Primary bile duct stones	48.936	8.658	276.596	<0.001
	Cholangitis without bile duct stones	9.857	1.19	81.62	0.034
Cholecystectomy*					
	Size				
	<1 cm	0.071	0.005	0.984	0.049
	≥1 cm and <3 cm	0.046	0.004	0.537	0.014

* Comparison with conservative treatment as a reference.

### Analysis of surgical indications for patients with symptomatic DD and the corresponding complications

To make the appropriate surgical choice for patients with symptomatic DD, we reviewed the records of all 64 patients who underwent surgery and attempted to summarize the potential surgical indications for each procedure (Table 5). Our results showed that choledochojejunostomy (28, 43.8%) was the most common procedure, followed by gastrojejunostomy (13, 20.3%), diverticulectomy (9, 14.1%), etc. Patients undergoing gastrojejunostomy and diverticulectomy appear to be more commonly combined with gallbladder stones and cholangitis. As for choledochojejunostomy and united surgery, patients were more likely to have a combination of cholangitis and bile duct dilatation. There was no clear preference for the other two types of surgery, probably because of the low cases.

**Table 5:** Analysis of surgical indications for patients with symptomatic diverticula.

	Gastrojejunostomy (*n* = 13)	Choledochojejunostomy (*n* = 28)	Diverticulectomy (*n* = 9)	Diverticula suture (*n* = 2)	Pancreaticoduodenectomy (*n* = 4)	United surgery^a^ (*n* = 8)
Diverticulitis	4	5	3		1	2
Gastrointestinal bleeding	1	1				
Digestive tract obstruction	2	1				
Gallbladder stones	6	13	7	1	1	5
Cholangitis	6	25	6	2	2	8
Bile duct dilatation	4	20	4	1	1	7
Bile duct pneumatosis		6			2	
Biliary stricture/obstruction	4	13	2		2	7
Pancreatic duct dilatation	1	2			2	1
Pancreatic duct stones	1	1			2	
Pancreatitis	6	2	1	1	3	2
Pancreatic pseudocysts					1	1
Intestinal fistula	1	2				
Biliary fistula		2				
Duodenal ampulla cancer					2	
Bile duct cancer		1			1	1

^a^ Includes 2 cases of the diverticula suture + choledochojejunostomy procedure, 4 cases of the diverticulectomy + choledochojejunostomy procedure, and 2 cases of the gastrojejunostomy + choledochojejunostomy procedure.

Encouragingly, the frequency of complications among patients who underwent surgical treatment was low (7/64, 10.9%). Complications are mainly from patients who have undergone gastrojejunostomy and choledochojejunostomy (Table 6), and most of them were middle-aged and elderly women. All complications were not fatal, as all patients were discharged in good condition after various conservative treatment measures, which suggests that surgical treatment of DD is safe and feasible.

**Table 6:** Postoperative complications and outcomes of patients undergoing surgical treatment.

Surgical procedure	Types of complications	Sex	Age	Outcomes
Gastrojejunostomy	Gastroparesis	Female	66	Survival
	Gastrointestinal bleeding	Male	51	Survival
	Intestinal fistula	Female	63	Survival
Choledochojejunostomy	Anastomotic fistula	Female	45	Survival
	Biliary fistula	Female	66	Survival
	Biliary bleeding	Female	59	Survival
Pancreaticoduodenectomy	Biliary fistula	Female	66	Survival

## Discussion

DD as a benign digestive tract disease is widely distributed in the population. Patients with DD are usually asymptomatic and therefore often receive insufficient attention (15), but in our study, more than half of the patients (53.3%) had a combination of various symptoms. Meanwhile, the most common clinical symptoms (such as epigastric pain, nausea, vomiting, and abdominal distension) of DD patients often lack specificity and are often combined with multiple symptoms at the same time, making them easy to misdiagnose in the clinic and preventing them from receiving appropriate treatment.

By comparing the differences in clinical characteristics between patients in the symptomatic and asymptomatic groups, we found that patients with DD located in periampullary and ≥1 cm seemed to be more likely to have clinical symptoms. It is well known that DD is more common in the 2nd portion and periampullary, and different hypotheses (16, 17) have also been proposed that DD in this area is more likely to be combined with various pathological states. Our results firstly provide the evidence of this association, meanwhile we suggesting a possible association between diverticular size and the presence of clinical symptoms.

Previous studies lacked a unified standard for the classification of DD size because it was difficult to accurately measure diverticular size even under endoscopy. Kim et al. (8) chose 1.5 cm and 3.0 cm as the judgment bounds, because the diameter of a commonly used endoscopic stone picking balloon was exactly 1.5 cm, and the size of PAD could be more accurately measured with this reference. Based on our precise measurements of all measurable imaging pictures, we chose 1 cm, 3 cm, and 5 cm as the measured value of diverticular size, then found that smaller DD with the size of <1 cm and 1–3 cm were the majority (78.0%). Interestingly, there were also lots of patients in our study who had no obvious clinical symptoms, so this phenomenon can be partially explained by the above conclusions, as we all know usually a small DD is less likely to cause symptoms because it has little effect on the primary physiological function.

Patients with DD usually have symptoms that do not arise directly from the diverticula, but rather from various types of diverticula-related complications and comorbidities. In addition to comorbidities such as diverticulitis, various complications related to the biliary system and the pancreas are also included. The association between DD and Biliopancreatic disorders has been confirmed by many previous studies. Karn et al. (18) recently completed a meta-analysis including 11 related studies and concluded that patients with PAD had a significantly increased risk of choledocholithiasis about 2.3 times that of normal people. Bruno et al. (19) conducted a 2,475 EUS examination on patients with PAD and showed that the prevalence of cholangitis, bile duct dilatation, and choledocholithiasis was significantly higher than those in the control group without DD.

In our study, patients with DD combined with different types of biliopancreatic comorbidities were significantly more common in PAD and DD >1 cm, again confirming our previous findings. As for the reasons for the association, there are several possible hypotheses in previous studies: (1) Mechanical pressure exerted by the diverticula on the distal portion of the common bile duct can impede bile excretion (20); (2) The diverticula may cause sphincter dysfunction of Oddi. It may be due to sphincter stenosis due to the accumulation of food or bezoar in the diverticula. Or chronic ampullary inflammation caused by diverticula can lead to chronic fibrosis of the nipple and subsequent stenosis (21); (3) Bile stasis and abnormal tension and contractile activity of Oddi sphincter may lead to the spread of overgrown bacteria in the diverticula to the biliary tract system more easily, and produce β-glucuronidase and debinding bile salts, thus forming stones (22, 23).

Given high proportion of patients in the symptomatic group in our study, the management of this group of patients deserves high attention. In our study, conservative treatment (65.2%) was predominant in the treatment of DD, which suggests that a high number of smaller-sized or single DD are not usually associated with a serious outcome. Depending on the type and severity of the complications, endoscopic treatment and cholecystectomy may be options for complications only, in addition to surgical treatments that directly target DD.

Our univariate analysis found that a greater number of clinical characteristics may be associated with the choice of treatment modality. Further, we performed a multivariate Logistics regression analysis and the results showed that patients with combined smaller-sized DD may prefer conservative treatment, while patients with combined biliary system stones and cholangitis without bile duct stones may prefer surgical treatment (direct or indirect). Previous studies on the treatment of patients with DD are scarce, and our findings will be very helpful in making decisions on the treatment for these patients.

The main surgical treatment modalities for patients with DD in previous studies (24) include diverticulectomy, duodenal resection, and diverticular inversion, and surgical treatment for DD is considered safe. In our study, DD patients were operated on more frequently with choledochojejunostomy (43.8%) and gastrojejunostomy (20.3%), next by diverticulectomy (14.1%). Based on a review of the indications for each procedure, we summarized the more frequent indications of each procedure for the reference of subsequent operators. As for postoperative complications, our results are consistent with those reported (24, 25), with a low probability of postoperative complications in diverticulosis patients (10.9%). Complications occur mostly in middle-aged and elderly female patients and are non-fatal, and all improved after conservative treatment.

As mentioned earlier, our study mainly focuses on two questions: What kind of DD patients deserve attention and management; and what kind of management is appropriate for this type of DD patient? Although it was a retrospective study, we suggest possible explanations by analyzing a large number of case data. Our study is also the only detailed and in-depth study done to date on the evaluation and management of DD.

Still, our study needs to be improved in the following aspects: groups based on DD size may be slightly biased from the true situation because we cannot ensure that the data measured by the electronic caliper is completely accurate, especially when it is influenced by the diverticular contents. More importantly, limited by the nature of retrospective studies, our answers to the two questions can only provide possible interpretations. As for the exact causal relationship, further confirmation is needed in subsequent multicenter, prospective studies.

In conclusion, DD is a common clinical pathology frequently occurring in the 2nd portion, mostly small in size and single in number. Patients with DD ≥1 cm or located in the periampullary are more likely to be combined with various types of comorbidities and complications, thus presenting as symptomatic. The size of the DD and the combination of specific biliary comorbidities may have an impact on the choice of treatment modality. Although most patients with symptomatic DD can be treated conservatively only, surgical treatment is also a safe and effective approach when the appropriate procedure is chosen. Our findings will provide important ideas for the clinical diagnosis and treatment of DD, but further prospective studies are needed to confirm that.

## Data Availability

The raw data supporting the conclusions of this article will be made available by the authors, without undue reservation.
