# Supplementary figures and images for: Evaluation and management of symptomatic duodenal diverticula: a single-center retrospective analysis of 647 patients
Source: Front Surg. 2023 Aug 30;10:1267436. doi: 10.3389/fsurg.2023.1267436 (PMC10498277; doi:10.3389/fsurg.2023.1267436)

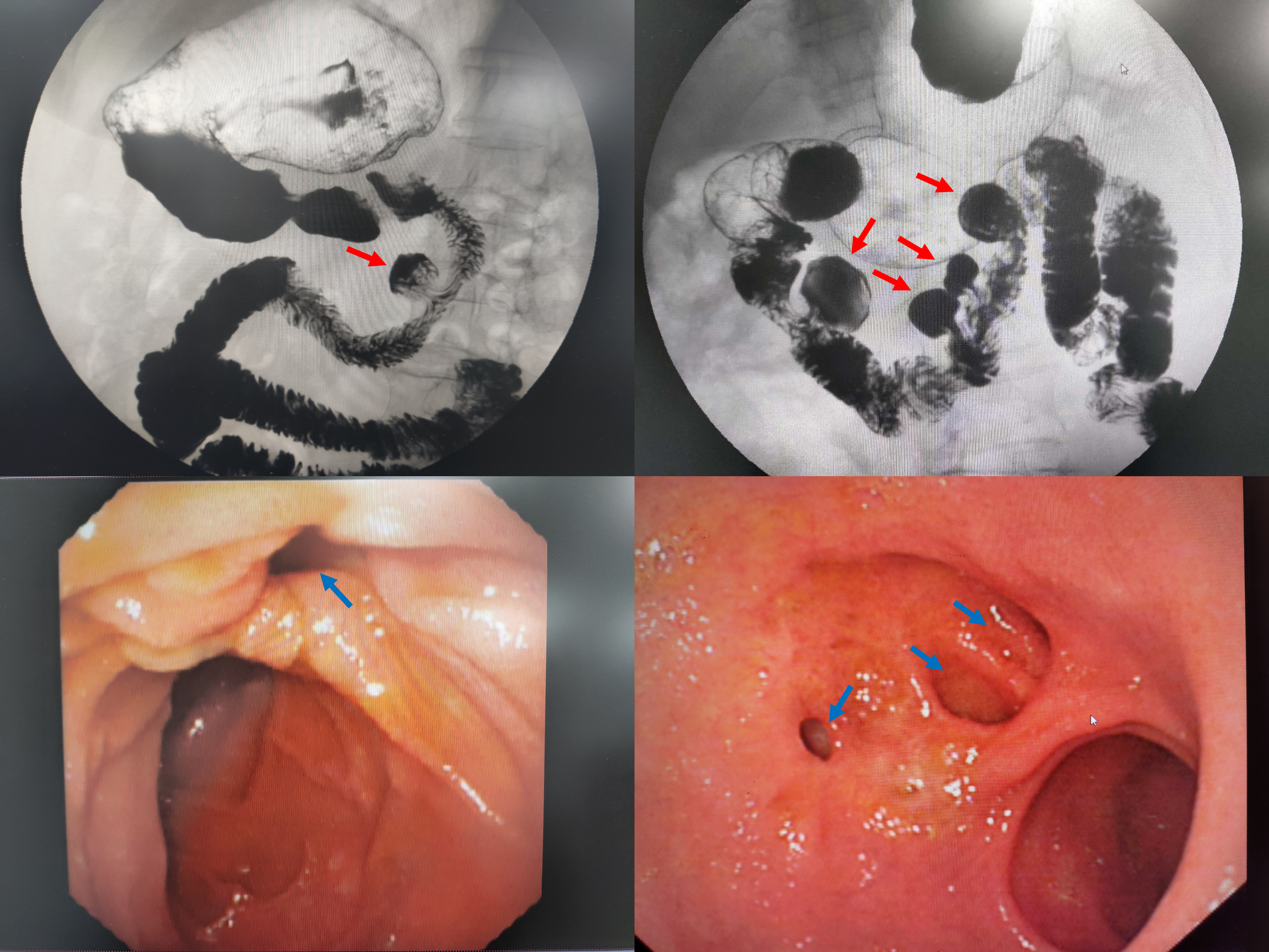

Supplement: Supplementary file 1 [file Image1.tif]
